# Improving Access to Treatment for Opioid Use Disorder in High-Need Areas: The Role of HRSA Health Centers

**DOI:** 10.13023/jah.0204.04

**Published:** 2020-09-01

**Authors:** Michael Topmiller, Jennifer Rankin, Jessica L. McCann, Jene Grandmont, David Grolling, Mark Carrozza, Hank Hoang, Josh Bolton, Alek Sripipatana

**Affiliations:** HealthLandscape, American Academy of Family Physicians; U.S. Department of Health and Human Services, Health Resources and Services Administration

**Keywords:** Appalachia, medication-assisted treatment (MAT), opioid-use disorder, health centers, geospatial analysis, rural health

## Abstract

**Introduction:**

Despite the opioid epidemic adversely affecting areas across the U.S. for more than two decades and increasing evidence that medication-assisted treatment (MAT) is effective for patients with opioid use disorder (OUD), access to treatment is still limited. The limited access to treatment holds true in the Appalachia region despite being disproportionately affected by the crisis, particularly in rural, central Appalachia.

**Purpose:**

This research identifies opportunities for health centers located in high-need areas based on drug poisoning mortality to better meet MAT care gaps. We also provide an in-depth look at health center MAT capacity relative to need in the Appalachia region.

**Methods:**

The analysis included county-level drug poisoning mortality data (2013–2015) from the National Center for Health Statistics (NCHS) and Health Center Program Awardee and Look-Alike data (2017) on the number of providers with a DATA waiver to provide medication-assisted treatment (MAT) and the number of patients receiving MAT for the U.S. Several geospatial methods were used including an Empirical Bayes approach to estimate drug poisoning mortality, excess risk maps to identify outliers, and the Local Moran’s I tool to identify clusters of high drug poisoning mortality counties.

**Results:**

High-need counties were disproportionately located in the Appalachia region. More than 6 in 10 health centers in high-need counties have the potential to expand MAT delivery to patients.

**Implications:**

The results indicate an opportunity to increase health center capacity for providing treatment for opioid use disorder in high-need areas, particularly in central and northern Appalachia.

## INTRODUCTION

Despite the opioid epidemic adversely affecting areas across the U.S. for more than 2 decades^1^ and increasing evidence that medication-assisted treatment (MAT) is effective for patients with opioid-use disorder (OUD), access to treatment is still limited.^2^ The lack of access to treatment holds true in the Appalachia region, despite the region having been disproportionately affected by the crisis, particularly in rural, central Appalachia.^3^ While the opioid epidemic is a primary contributor to higher mortality rates in Appalachia,^4^,^5^ both improving access to comprehensive health care and evidence-based treatments (such as MAT) have been identified as ways for addressing the opioid crisis in the region.^6^

Health Resources and Services Administration (HRSA)–funded health centers and Health Center Program Look-Alikes were included in this analysis to explore ways to better address a growing OUD care need. HRSA-funded health centers are community-based and patient-directed organizations receiving funding through Section 330 of the Public Health Service Act. Health Center Look-Alikes do not receive Health Center Program funding but operate and provide services consistent with the Health Center Program requirements.^7^ Both HRSA-funded health centers and look-alikes (henceforth referred to as health centers) provide comprehensive primary care services in underserved areas and integrate access to pharmacy, mental health, substance use disorder, and oral health services in areas where economic, geographic, or cultural barriers limit access to affordable healthcare services.^8^

With 1373 health centers serving more than 27 million patients in 2017, health centers are fundamentally and geographically well-positioned to address the lack of access to treatment, as they focus on vulnerable populations and integrating access to primary care, pharmacy, mental health, and substance abuse services. Many health centers have Drug Addiction Treatment Act (DATA)–waived providers, who are licensed to prescribe buprenorphine as part of MAT (henceforth referred to as health center MAT capacity).^9^ However, research is needed to better understand current health center MAT capacity in high-need areas. This research identifies health centers located in high-need areas based on drug poisoning mortality and explores their capacity to provide MAT. An in-depth look at the Appalachian region is also provided, and at the capacity of health centers to provide MAT in high-need areas within Appalachia.

## METHODS

The analysis included county-level drug poisoning mortality data (2013–2015) from the National Center for Health Statistics (NCHS),^10^ county economic status data from the Appalachian Regional Commission (ARC, FY2019),^11^ and Health Center Program Awardee and Look-Alike data (2017 Uniform Data System)^12^ on the number of providers with a DATA waiver to provide medication-assisted treatment and the number of patients receiving MAT^9^ as a proxy for capacity to provide OUD care.

All participating organizations of the Health Center Program are required to report standardized annual data to HRSA through the Uniform Data System (UDS). Included in the UDS are data on patient demographics, performance in clinical quality measures, staffing and service utilization, and financial data, as well as the number of physicians, certified nurse practitioners, and physician assistants, on-site or with whom the health center has contracts, that have obtained a DATA waiver to treat opioid-use disorders with medications approved by the U.S. Food and Drug Administration. Health centers also report the number of patients who received MAT for opioid-use disorder from one of these providers. It is important to note that health center data are reported at the administrative level, meaning while each of the health centers may have multiple health center delivery sites, only the administrative locations (which often serve as health center delivery sites as well) are being mapped.

High-need counties were defined based on drug poisoning mortality (ICD-10 Codes: X40–X44, X60–X64, Y10–Y14) using GeoDa software^13^ and involved multiple steps, including the following: first, counties with zero deaths were removed from the analysis; next, estimates of drug poisoning mortality death rates were created using an Empirical Bayes (EB) approach, which adjusts rates based on population numbers towards the overall mean (counties with small populations are adjusted more); next, excess risk maps were created to identify counties with adjusted drug poisoning death rates 1.5 times the national average; finally, the Local Moran’s I tool was used to identify statistically significant clusters of counties (hot spots) with high adjusted rates of drug poisoning mortality.^13^ A county was defined as high-need if it has a drug poisoning mortality excess risk of 1.5 times the national average or is part of drug poisoning mortality hot spot.

After identifying high-need counties, health centers were mapped and stratified by current and potential capacity for MAT (based on their number of DATA waived providers and patients receiving MAT). Current and potential health center capacity was explored based on their number of DATA-waived providers and number of patients receiving MAT. Health centers with at least one DATA-waived provider but had no patients receiving MAT were also highlighted. Note that health center MAT capacity is defined by the number of providers with a DATA waiver to prescribe buprenorphine as a part of MAT and does not include health centers that only provide other kinds of medications as part of MAT (i.e., naltrexone and methadone).

## RESULTS

**Figure 1:**
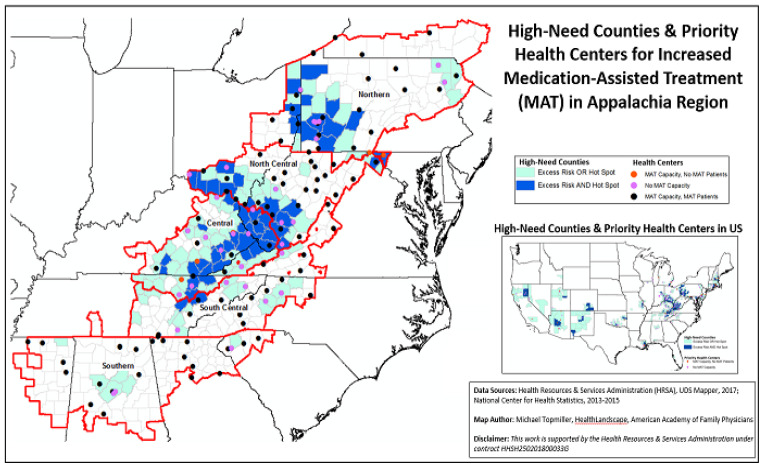
High-Need Counties and Health Center MAT Capacity, Appalachia

Figure 1 displays the 368 high-need counties, which have significantly higher drug poisoning mortality rates than non–high-need counties (25.8 per 100,000 vs. 15.2 per 100,000) and are clustered in several areas across the U.S. The darker colored counties (which meet both criteria of excess risk and hot spot) are usually the central counties in large high drug poisoning mortality clusters. Most high-need counties have at least one health center with MAT capacity, though this varies by geography with a lack of health center MAT capacity in some areas, including parts of the southwestern U.S. and Appalachia.

About one fifth (19.6%) of all 1429 health centers are located in high-need counties, with a little more than one half (55.5%) of these having at least one provider with a DATA-waiver, and 44.1% having patients utilizing MAT. More than one fifth of the health centers with at least one DATA-waived provider do not have any patients utilizing MAT.

Consistent with literature documenting higher rates of mortality in Appalachia, Figure 1 shows that high-need counties are disproportionately located (40% of all high-need, 35% of all Appalachia counties) in the Appalachia region, including more than half of all counties that were identified as both drug poisoning mortality excess risk and hot spots. The largest number of high-need counties are in Central Appalachia (43.2% of high-need counties, 78.0% of all counties located in Central Appalachia), though many are also located in North Central and Northern Appalachia (39.2%). High-need Appalachia counties are also more likely to have an economic status of distressed, defined by the ARC as counties in the bottom quintile based on a composite measure of unemployment, per capita income, and poverty.^11^

Compared to nationally, a lower proportion of the 137 health centers located in Appalachia are providing MAT, where more than 6 in 10 (63.5%) have no DATA-waived provider and more than 7 in 10 (70.8%) have no MAT patients, compared to 58.9% and 67.0% for the U.S., respectively. There are 151 DATA-Waived providers in 50 health centers in Appalachia serving 4440 MAT patients. Almost one half of health centers in Appalachia are located in high-need counties. More than one half (54.4%) of these health centers have no MAT providers and more than 6 in 10 (61.7%) did not report providing MAT to its patients. More than 3000 MAT patients are being seen by 96 DATA-waived providers at 31 health centers in high-need counties.

## IMPLICATIONS

The results indicate an opportunity to increase health center capacity for providing treatment for OUD in the Appalachia region. For example, health centers in high-need counties that did not indicate MAT capacity at the time the data were reported could be targeted for future outreach and resource allocation to increase number of DATA-waived providers; while health centers with MAT capacity but no MAT patients could be targeted for research and funding to identify reasons current DATA-waived providers are not providing MAT and provide incentives for treating more patients. This additional funding and research should also address the role of stigma in reducing access to treatment.^14^ As health centers already play an important role in improving access to health care in the Appalachia region, improving their capacity to deliver MAT could have a major impact in alleviating the opioid crisis. Indeed, increasing access to evidence-based treatment is one of recommendations of several reports focused on Appalachia, along with expanding telehealth services for treatment of opioid-use disorder, which is another way area health centers are well-suited to contribute.^6^

While the intent of this research is to identify opportunities to better respond to opioid-use disorder in Appalachia by leveraging health centers’ capacity, it has limitations related to data availability. First, high-need counties were defined using drug-poisoning mortality, which includes non-opioid drugs. Second, the mapping exercise included health centers as opposed to health center delivery sites. A health center entity can have multiple delivery sites, which can be located in different counties, and it is not possible to determine at which sites MAT is delivered since data in the UDS are reported at the parent/network organization level. Not including delivery sites in the analysis likely leads to an underestimation of health center capacity in the region. A third, related limitation is that we explored health center capacity in Appalachia focusing only on health centers located within Appalachia and did not include health centers that were located outside of Appalachia but had delivery sites within Appalachia. An additional limitation that may have led to underestimating health center capacity is that data for health centers providing MAT using naltrexone or methadone are not available.^15^ Finally, data for other providers unaffiliated with HRSA health centers were not included in this analysis. These non-health center MAT providers may introduce competitive market forces and present barriers for health centers from entering the service area. Future research should look to incorporate state/local opioid-specific mortality data along with more detailed data on MAT providers (both from health center delivery sites and other DATA-waived providers) in the region.

**SUMMARY BOX**

**What is already known about this topic?**

**What is added by this report?** Health centers are well-positioned to address opioid epidemic, but health centers in high-need areas, particularly in Central Appalachia, lack treatment capacity.

**What are the implications for future research?**

This research identifies priority health centers that could be targeted for expanded resources to improve MAT capacity in high-need counties in Appalachia.

## References

[1] Rossen LM Bastian B Warner M Khan D Chong Y Drug poisoning mortality: United States, 1999–2017 Hyattsville (MD) National Center for Health Statistics 2019 https://www.cdc.gov/nchs/datavisualization/

[2] National Academies of Sciences, Engineering, and Medicine Medications for opioid use disorder save lives Washington The National Academies Press 2019 10.17226/25310 30896911

[3] Moody L Satterwhite E Bickel WK Substance abuse in rural central Appalachia: Current status and treatment considerations Rural Ment Health 2017 41 2 123 135 10.1037/rmh0000064. 29057030 PMC5648074

[4] Woolf SH Schoomaker H Hill L Orndahl CM The social determinants of health and the decline in U.S. life expectancy: implications for Appalachia J Appalach Health 2019 1 1 6 14 10.13023/jah.0101.02. PMC9173599 35769540

[5] Scutchfield FD Root causes of Appalachia’s deaths of despair J Appalach Health 2019 1 2 1 6 10.13023/jah.0102.01. PMC9138851 35769901

[6] East Tennessee State University and NORC at the University of Chicago Health disparities related to opioid misuse in Appalachia Washington Appalachia Regional Commission 2019 https://www.arc.gov/assets/research_reports/HealthDisparitiesRelatedtoOpioidMisuseinAppalachiaApr2019.pdf

[7] Health Resources and Services Administration Health Center Program Look-Alikes website https://bphc.hrsa.gov/programopportunities/lookalike/index.html

[8] Health Resources and Services Administration What is a Health Center website https://bphc.hrsa.gov/about/what-is-a-healthcenter/index.html

[9] Health Resources and Services Administration Health Center Data & Reporting website https://bphc.hrsa.gov/datareporting/index.html

[10] National Center for Health Statistics Drug poisoning mortality 2013–2015 as compiled from data provided by the 57 vital statistics jurisdictions through the Vital Statistics Cooperative Program [Data file] https://www.cdc.gov/nchs/index.htm

[11] Appalachian Regional Commission County Economic Status and Distressed Areas in Appalachia [Data file] https://www.arc.gov/appalachian_region/CountyEconomicStatusandDistressedAreasinAppalachia.asp

[12] Health Resources and Services Administration UDS Mapper website https://www.udsmapper.org

[13] Anselin L Syabri I Kho Y GeoDa: An Introduction to Spatial Data Analysis Geogr Anal 2016 38 1 5 22 10.1111/j.0016-7363.2005.00671.x.

[14] Richard EL Schalkoff CA Piscalko HM “You are not clean until you’re not on anything”: Perceptions of medication-assisted treatment in rural Appalachia Int J Drug Policy (Article in Press) 10.1016/j.drugpo.2020.102704 PMC8018539 32173274

[15] Corallo B Tolbert J Sharac J Markus A Rosenbaum S Medication-Assisted Treatment for Opioid Use Disorder Kaiser Family Foundation 2020 https://www.kff.org/uninsured/issue-brief/community-healthcenters-and-medication-assisted-treatment-for-opioid-usedisorder/view/footnotes/

